# Infection caused by *Lawsonella clevelandensis* after breast augmentation with autologous fat grafting: a case report

**DOI:** 10.1186/s12879-022-07812-6

**Published:** 2023-02-28

**Authors:** Feng Zhou, Jun Zhang, Lunli Gong, Guobao Wang, Aawrish Khan, Haiyan Cui

**Affiliations:** Department of Plastic and Aesthetic Surgery, Tongji Hospital Affiliated to Tongji University, No. 389, Xincun Road, Putuo District, Shanghai, 200065 China

**Keywords:** Autologous fat grafting, Acid-fast pathogenic bacterium, Infection, *Lawsonella clevelandensis*, Case Report

## Abstract

**Background:**

*Lawsonella clevelandensis* is one recently documented anaerobic, which is partially acid-fast. Nevertheless, it is rarely found to be associated with human infections, especially in scope of plastic and cosmetic surgery before our patient who was performed breast augmentation with autologous fat grafting. Breast augmentation is becoming popular, the most common post-surgery complication of which is bacterial infection.

**Case presentation:**

A 29-year-old female who was found swelling in her right breast and fever after breast augmentation surgery with autologous fat grafting was administered. Before administration, she had been treated with antibiotics (details unknown) for more than 1 month without any significant improvements. After administration, she was treated with intravenous antibiotic empirically and repeated debridement via Vaccuum Sealing Drainage (VSD). And samples of the necrotic tissues and pus collected in surgery were sent for microbiological testing. However, routine examination failed. Thus samples were further collected and sent to Genoxor Medical & Science Technology Inc. (Shanghai, China) to conduct Next-Generation Sequencing (NGS). Surprisingly *Lawsonella clevelandensis* was determined. Accordingly, sensitive antibiotic was applied in concert with thorough debridement and drainage and finally her condition was completely reversed with wound closure gradually.

**Conclusion:**

Complications of breast augmentation with autologous fat graft are various, of which infection is most common. Rare pathogen such as *Lawsonella clevelandensis* infection in human is rare in clinical practice. Moreover, it is difficult to differentiate from non-tuberculous mycobacterium for its partial acid resistance, difficulty to culture and abscess formation. How to determine diagnosis of *Lawsonella clevelandensis* infection accurately come to be critical In our report, NGS is recommended as a useful method to identify the pathogen, which may provide us a novel tool for refractory wound.

## Background

Breast augmentation with autologous fat is a popular cosmetic surgery performed all over the world currently [1]. However, post-surgery complications are various, of which bacterial infection is the most common [2].

Recently, our department admitted one patient suffering from refractory wound which was finally diagonosed as *Lawsonella clevelandensis* infection after breast augmentation with autologous fat grafting. Although detailed information and nomenclature of *Lawsonella clevelandensis* was published in 2016 [3, 4], case of postoperative *Lawsonella clevelandensis* infection following autologous fat transplantation has not yet reported to our knowledge so far.

## Case presentation

A 29-year-old female underwent breast augmentation with autologous fat grafting at one clinic in Shanghai, China. And swelling of the right breast was observed being more severer on the 2nd day postoperation. Initially the swelling was considered as post-surgery swelling, therefore no special attention was taken. Unexpectedly critically severe swelling on the right side showed with high fever, which running up to 39.5 °C.

Then she was then transferred to another clinic and treated with intravenous Ceftriaxone, 3.0 g, q8h for 2 weeks. Unfortunately, the redness, swelling and pain in her right breast persisted without any improvement tendency.

Subsequently incision and drainage of the right breast was carried out under local anesthesia, meanwhile antibiotics were changed to empirical use of intravenous Levofloxacin, 1.0 g, bid. Still no improvement tendency showed.

Then she was transferred to a Grade A hospital in Shanghai being diagnosed as “soft tissue infection” and antibiotics was adjusted to “intravenous penicillin” (more detailed information is unknown). Nevertheless no sign of significant improvement turned up. Finally she was then hospitalized in our department, on the 58th day after surgery.

**Fig. 1:**
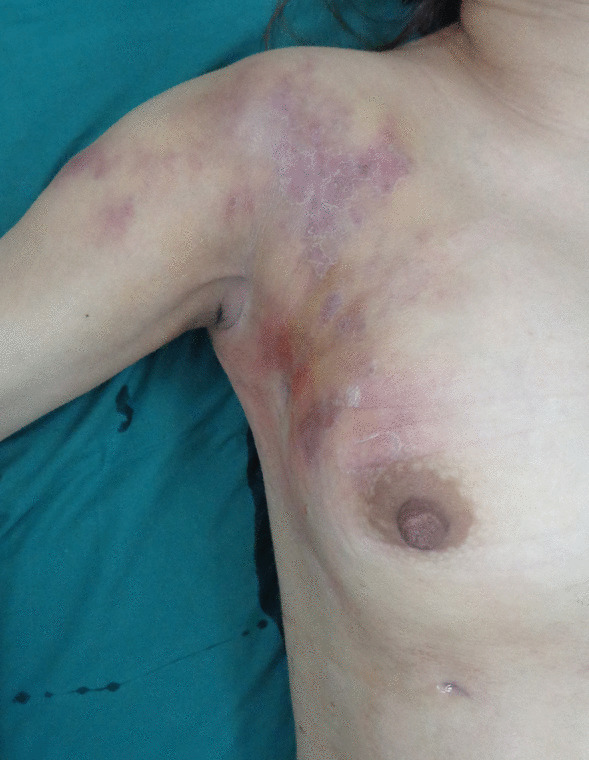
Obvious redness and swelling ranging from the right breast to right chest and shoulder were detected, accompanied with mottled bruise. Previous incision could be seen in the infra-mammary fold and the upper pole of the right breast

Upon admission, physical examination was performed and listed below: low fever being 37.3 °C, pulse being 107 bpm, respiratory rate being 20 bpm and blood pressure being 102/75 mmHg. And redness and swelling were observed ranging from the right breast to right chest. Moreover shoulder accompanied with mottled bruise was obvious. Besides, previous incisions in the infra-mammary fold and the upper pole of her right breast were notable with tendency to form fistula for visible pus outflowing. In addition, several abscesses beneath were obvious with the diameter being 10 cm maximally, and the foci temperature being elevated significantly. Surprisingly, several kermesinus scleroid blotches with higher skin temperature and distinct boundary from surrounding normal skin was found scattered around the pretibial area in both lower extremities (Fig. 1).

**Fig. 2:**
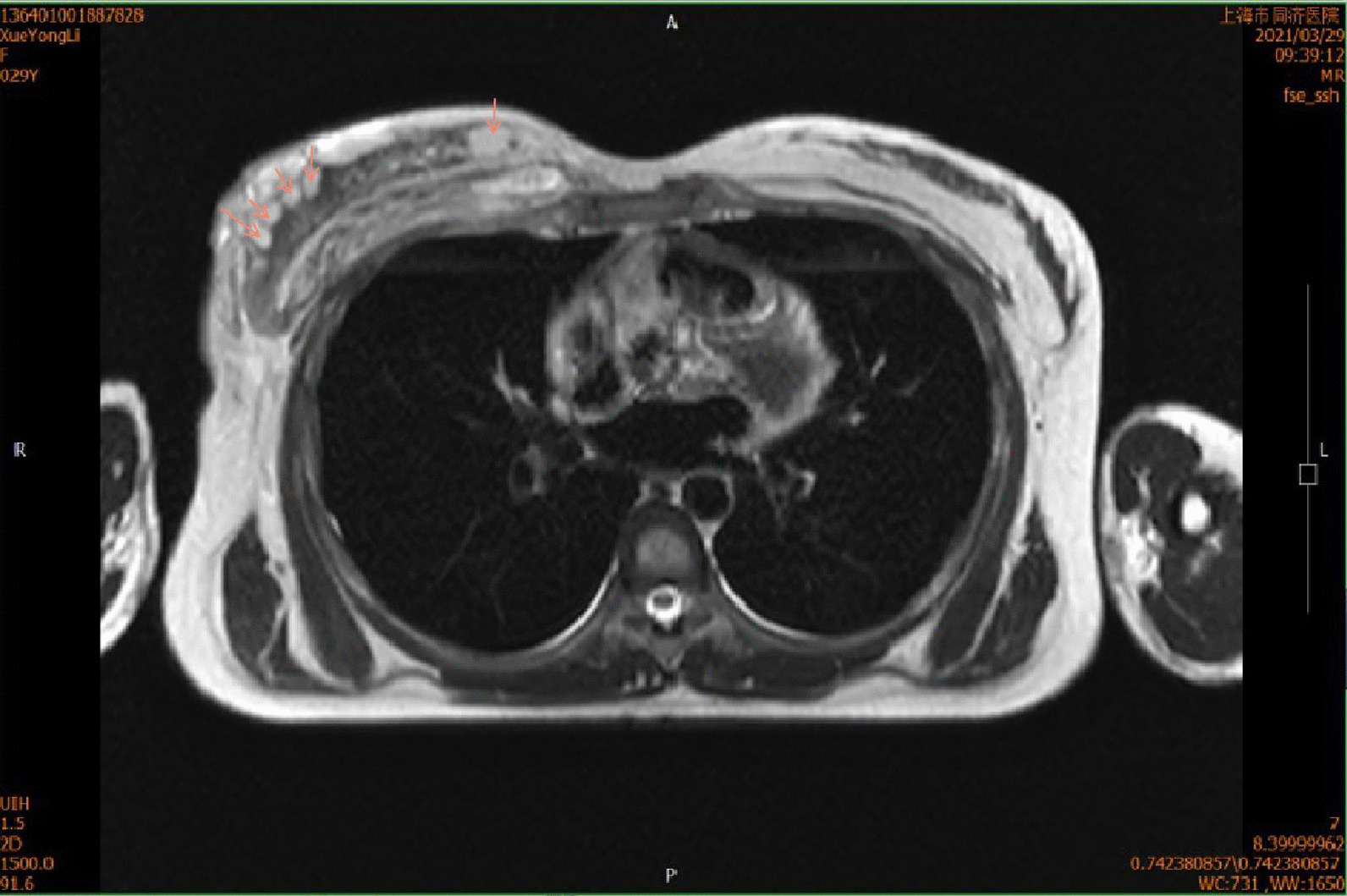
Magnetic Resonance Imaging (MRI) demonstrated: High signal intensity on T2-weighted images involving soft tissues in the region of the right pectorals major muscle, right shoulder and right upper arm was detected; multiple nodular-like high signal intensity (→) were shown in right mammary gland, which became hypointensity after lipid suppression

After admission, Magnetic Resonance Imaging (MRI) was conducted immediately. It was shown that pectorals major muscle, shoulder and upper arm in right side were sweeped by infection. Besides, multiple nodular-like foci were detected across the right breast (Fig. 2).

Moreover, apparent swollen soft tissues around the right shoulder joint capsule were perceived, which was the infected grafted fat. In addition, soft tissue swelling and infection could be detected via MRI of the knee (right). While the results of blood routine (BRT) are shown in Table 1.

**Table 1:** Blood RT before surgery

	D0	D2
WBC (× 10^9^)	11.44	10.99
N%	73.70	70.60
L%	20.40	18.20
N# (× 10^9^)	8.43	7.76
MONO# (× 10^9^)	0.64	1.14
CRP (mg/L)	42.74	62.16
PCT (ng/mL)	0.042	

Additionally, Piperacillin & Tazobactam and Levofloxacin was delivered intravenously after admission to cover both Gram-positive (G** +**) and Gram-negative (G −) bacteria as empirical antibiotic application, which would consequently be adjusted following microbiological studies afterwards. A total of 8 pus swab samples were collected for bacterial smear test, acid-fast stain, regular and anaerobic bacterial culture while blood samples for Beta-d-glucan fungal and TB-spot testing. However, no clues were obtained. Unaccountably the maximum temperature of the patient monitored daily fluctuated between 37.8 °C and 39.2 °C.

Sequentially, surgery for incision, exploration and debridement of the lesion was carried out repeatedly, additonally vacuum sealing drainage (VSD) was used to ensure thorough drainage. Meanwhile subcutaneous necrotic tissue and pus were collected to be sent to Genoxor Medical & Science Technology Inc. (Shanghai, China) for next-generation sequencing (NGS) as last straw, which was conducted under the protocol of NGS by the testing agency as previous similarly [5, 6].

Undisappointedly, the NGS results showed that *Lawsonella clevelandensis* infection was present in both pus and necrotic tissues, with the abundance being 96.60% and 100% respectively, while the sequence numbers were 141 and 6, respectively (Tables 2, 3, 4).

**Table 2:** Blood RT after 1st and 2nd Debridement

	D0 (1st Debridement)	D3	D8	D10 (2 days after the 2nd Debridement)	D14	D21
WBC (× 10^9^)	11.86	11.85	16.65	14.98	8.51	5.72
N%	72.80	70.80	77.60	76.80	68.9	40.5
L%	18.00	18.10	14.10	14.10	24.3	42.3
N# (× 10^9^)	8.63	8.39	12.92	11.50	5.86	2.32
MONO# (× 10^9^)	1.03	1.13	1.23	1.27	0.5	0.79
CRP (mg/L)	74.68	54.45	60.47	96.02	51.65	24.13
PCT (ng/mL)	0.031	0.030	0.045	0.034	0.032	0.029

**Table 3:** NGS high-throughput sequencing results of pus samples

Genre	Category	Sequence number	Breed	Sequence number	Relative abundance
G +	*Lawsonella*	141	*Lawsonella clevelandensis*	141	96.60%
	*Staphylococcus*	19	*Staphylococcus aureus*	3	1.36%

G + Gram-positive bacterium =, G − = Gram-negative bacterium

**Table 4:** NGS high-throughput sequencing results of tissue samples

Genre	Category	Sequence Number	Breed		Relative Abundance
G +	*Lawsonella*	6	*Lawsonella clevelandensis*	6	100%

G + Gram-positive bacterium =, G − = Gram-negative bacterium

**Fig. 3:**
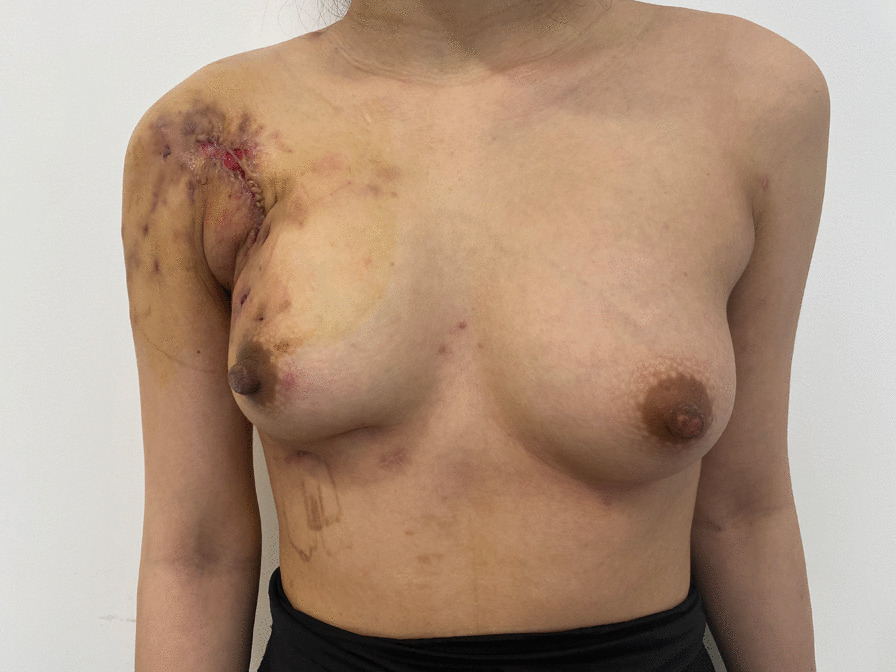
The residual wound was closed by direct suture to shorten the duration and narrow the wound. Deformity in her right breast, and asymmetry was obviously observed

Correspondingly, the antibiotics were adjusted to intravenous Cefoxitin (2.0 g, tid) and Ornidazole (500 mg, bid) following an adequate wound drainage and a thorough literature review [7–9]. Delightedly, her fever, white blood cells (WBC) and C-reactive protein (CRP) examination were significantly improved after therapy adjustment (Table 2). The residual wound on the right chest wall was limited and tended to close gradually. However, obvious deformity and asymmetry in her right breast were observed during 2 weeks postoperative follow up (Fig. 3).

## Discussion and conclusions

Breast augmentation with autologous fat injection is now popular throughout the world. However, postoperative complications deserve attention, especially infection after graft [1, 2] which is reported as high as 1%. And the mainly involved pathogens are dermal colonization bacteria, such as *Staphylococcus aureus* and *Staphylococcus epidermidis.* Unusual pathogens such as nontuberculous mycobacterial (NTM) need more attention [10].

*Lawsonella clevelandensis* is an anaerobic and partially acid-fast bacillus, which was taxonomically described recently and was considered a new species within a new genus in the suborder *Corynebacterineae* [3]. It is found to colonize in nasal cavity as well as hair follicles and regarded as a kind of skin colonization bacteria [11–13]. Furthermore, *Lawsonella clevelandensis* was reported as the most common bacterium identified both below the infundibulum and on the scalp surface [9, 13]. Given the fact that the bacterium could cause abscesses, it is more likely an opportunistic pathogen.

To our knowledge, there are only 12 cases of *Lawsonella clevelandensis* infection documented worldwildly as of now. Actually it is very difficult to be cultivated in regular manner, so determination of its infection is prone to be misdiagnosed [3, 7], which could justify the rare condition of *Lawsonella clevelandensis* infection to some extent. Gene sequencing analysis is recommended to help diagnosis [3], of which detection of 16S rRNA sequence is the main evidence for *Lawsonella clevelandensis* infection determination d [9, 14–16].

As documented, *Lawsonella clevelandensis* could cause infection of enterocoelia, spine and breast, showing a tendency to generate abscesses [2, 3, 14–17], which shared a similar course with our case, i.e. time-consuming and efforts-consuming to discriminate the pathogen.

Interestingly, the clinical manifestations of *Lawsonella clevelandensis* infection resembles that of Nocardia/NTM [14, 15, 17], i.e. acid-fast bacillus, abscess-formation and difficult to be cultivated in vitro. While NTM is now attracting increasing attention in plastic and aesthetic surgery [2, 10]. Since the treatment of NTM infection involves prolonged multidrug regimens, which is characteristic of consuming both medically and economically. Therefore differential diagnosis of *Lawsonella clevelandensis* infection from NTM should be emphasized, correspondingly NGS could be competent.

In terms of antibiotic treatment, Goldenberger et al. reported that *Lawsonella clevelandensis* was sensitive to most of commonly used antibiotics in vitro, including penicillin, gentamicin, levofloxacin, ceftriaxone, cefuroxime, piperacillin, etc. [4]. However, it was also declared to be an opportunistic pathogen and might gain extensive drug resistance after exposure to broad-spectrum antibiotics. Therefore, it was not suggested to rely on high-grade or broad-spectrum antibiotics application [3, 7, 9]. And thorough surgical intervention should be performed as early as possible, and supplemental VSD may be better to ensure enough drainage [10]. Otherwise, once massive tissues were involved as in our case, prognosis definitely would be bleak.

In conclusion, *Lawsonella clevelandensis* infection should be taken into consideration, especially in cases with continuous infection who showed little or no effect after autologous fat graft following broad-spectrum/long-term antibiotics application. Currently NGS is the main method to identify *Lawsonella clevelandensis* infection in supplementary of earlier surgical intervention and proper antibiotic therapy, both are necessary and effective to treat the infection.

## Data Availability

The datasets used and/or analyzed during the current study are available from the corresponding author on reasonable request.

## Abbreviations

BRT: Blood regular test
WBC: White cell count
N: Neutrophil
L: Lymphocyte
MONO: Monocyte
CRP: C reacted protein
PCT: Procalcitonin
MRI: Magnetic resonance imaging
VSD: Vacuum sealing drainage
NGS: Next generation sequence
NTM: Non-tuberculous mycobacteria
